# ﻿Complete mitochondrial genome of *Guigarracailaoensis* Wang, Chen & Zheng, 2022 (Cypriniformes, Cyprinidae) and its phylogenetic implications

**DOI:** 10.3897/zookeys.1190.113808

**Published:** 2024-01-22

**Authors:** Lan-Ping Zheng, Ying-Min Geng

**Affiliations:** 1 College of Chinese Materia Medica, Yunnan University of Chinese Medicine, 1076 Yuhua Road, Kunming 650500, Yunnan, China Yunnan University of Chinese Medicine Kunming China

**Keywords:** Illumina, Labeoninae, phylogeny, southwestern China

## Abstract

*Guigarracailaoensis* is a member of family Cyprinidae, subfamily Labeoninae (Cypriniformes) which was recently discovered in southwestern China. Following its initial description, additional information on this species has remained notably scarce. In the current study, we assemble the complete mitochondrial genome (mitogenome) of *G.cailaoensis* using the Illumina sequencing platform. The mitogenome is identified as a circular, double-stranded DNA sequence of 16,593 base pairs, encompassing 13 protein-coding genes (PCGs), 22 transfer RNA genes, two ribosomal RNA genes, and a putative control region. Maximum-likelihood and Bayesian-inference approaches were used to construct phylogenetic trees for three datasets: (i) PCG sequences of the complete mitogenome (dataset 1); (ii) PCG sequences of the complete mitogenome combined with nuclear DNA (ncDNA) (*Rag1*) sequence (dataset 2); and (iii) ncDNA (*Rag1*) sequences (dataset 3). Phylogenetic analyses position *G.cailaoensis* as a sister taxon to the lineage consisting of *Paraqianlabeolineatus* Zhao, Sullivan, Zhang & Peng, 2014 and *Pseudogyrinocheilusprochilus* Fang, 1933 in dataset 1, and to *Pseudogyrinocheilusprochilus* in dataset 2, species lacking an oral disc on the lower lip. However, *G.cailaoensis* showed a close relationship to the lineage consisting of *Discogobio* and *Discocheilus* in dataset 3, species possessing an oral disc on the lower lip. Nonetheless, a variety of species with an oral disc on the lower lip are clustered into different lineages across the three datasets that may indicate that the development of the oral disc is homoplastic within the subfamily Labeoninae. The outcomes of this study have the potential to support conservation efforts for this species and to enrich our understanding of genetic resources in the area.

## ﻿Introduction

*Guigarracailaoensis* Wang, Chen & Zheng, 2022, a recently described genus and species in the subfamily Labeoninae of the family Cyprinidae (Cypriniformes), is a small fish adapted to torrent-water environments. To date, it has only been recorded in a small tributary of the Hongshuihe River in Guangxi Province, China. There it inhabits small streams in the upper reaches of the tributary, while being notably absent from the lower reaches (Wang et al. 2022). These environments are very fragile, rendering *G.cailaoensis* a potential indicator of local ecological conditions.

The subfamily Labeoninae, recognized by its unique oral morphology (Zhang et al. 2000), comprises more than 40 genera and 500 species. Within this subfamily, eight genera and nearly 200 species are characterized by a structurally varied oral disc on the lower lip (Wang et al. 2022). *Guigarra* Wang, Chen & Zheng, 2022, which also exhibits this feature, is a recently discovered genus found in the karst region in southwestern China, following the discovery of *Lanlabeo* Yao, He & Peng, 2018 in the same area.

Previous research on the subfamily Labeoninae has predominantly focused on taxonomy, particularly the description of new genera and species, as well as molecular phylogenetics. In recent years, various new Labeoninae genera and species from the karst region of southwestern China have been described, including *Sinigarra* Zhang & Zhou, 2012, *Paraqianlabeo* Zhao, Sullivan, Zhang & Peng, 2014, *Prolixicheilus* Zheng, Chen & Yang, 2016, *Zuojiangia* Zheng, He, Yang & Wu, 2018, *Lanlabeo* Yao, He & Peng, 2018, and *Guigarra* Wang, Chen & Zheng, 2022 (Zhang and Zhou 2012; Zhao et al. 2014; Zheng et al. 2016, 2018; Yao et al. 2018; Wang et al. 2022), highlighting the rich species diversity in this subfamily. Furthermore, molecular phylogenetic studies have elucidated the phylogenetic relationships within the subfamily and validated the classification of genera (Yang and Mayden 2010; Zheng et al. 2010, 2012; Yang et al. 2012). Yang et al. (2012) identified four primary clades within the subfamily Labeoninae, with Zheng et al. (2016) defining the karst group as part of the fourth clade. Wang et al. (2022) further established that *G.cailaoensis* belongs to this karst group.

Mitochondrial genomes (mitogenomes) are characterized by a simple molecular structure, strict maternal inheritance, minimal recombination, and a rapid evolutionary rate, making them valuable markers in studies of molecular population genetics and phylogenetics (Xiao and Zhang 2000). As mitogenomic research has advanced, the mitogenomes of fewer than 100 species of Labeoninae have been sequenced and deposited in GenBank. In this study, we successfully sequenced the complete mitogenome of *G.cailaoensis*. Our findings could contribute to the conservation of this species and further enrich genetic resources.

## ﻿Materials and methods

### ﻿Sample collection, DNA extraction, and quality testing

The sample used in this study was collected from the Cailaohe River, Fengshan, Guangxi, China (24.61°N, 106.97°E). Total genomic DNA was extracted from fin-tissue samples using a DNA isolation kit (Qiagen) with a final elution volume of 50 µl. The quality and purity of the isolated DNA were assessed prior to downstream applications. Agarose gel electrophoresis was used to analyze DNA integrity and assess the presence of contaminants. DNA purity was evaluated using a NanoDrop One spectrophotometer (Thermo Fisher Scientific, USA). Final DNA concentrations were accurately determined using a Qubit 3.0 Fluorometer (Thermo Fisher Scientific, USA).

### ﻿Library construction, mitogenome assembly, and annotation

The collected DNA sample was used for paired-end (PE) library construction using standard protocols of the NEBNext Ultra II DNA Library Prep Kit for Illumina (NEB, USA) in accordance with the manufacturer’s instructions. It was sequenced using the Illumina NovaSeq 6000 platform (Illumina, USA) with a 350-bp insert size. Adaptor and low-quality reads were filtered using fastp (Chen 2023), resulting in a total of 69.21 Mb of clean reads (150 bp). The mitogenome was *de novo* assembled using MitoZ (Meng et al. 2019). The assembled mitogenome was annotated using the online tool MITOS using the default parameters (Bernt et al. 2013). The protein-coding sequences were checked and confirmed using Geneious R10 (Kearse et al. 2012). Start/stop codons, codon usages, relative synonymous codon usage (RSCU), and nucleotide composition were analyzed using MEGA v. 7 (Kumar et al. 2016) and PhyloSuite (Xiang et al. 2023). Skew compositions were calculated using: AT-skew = (A − T) / (A + T) and GC-skew = (G − C) / (G + C) (Perna and Kocher 1995). TRNAscan-SE v. 2.0 (Lowe and Chan 2016) was used to predict the secondary structures and anticodons of transfer RNAs (tRNAs). The online mitochondrial visualization tool OGDRAW (Greiner et al. 2019) was used to draw a graphical map of the complete mitogenome. The newly generated complete mitogenome sequence and its annotation were submitted to GenBank using BankIt (accession number OR492308).

### ﻿Phylogenetic analysis

To determine the phylogenetic position of *G.cailaoensis*, 92 complete mitogenomes and 68 *Rag1* sequences of Labeoninae were downloaded from GenBank, and one species of Torinae, two species of Xenocypridinae, and three species of Opsariichthyinae were used as the outgroups (Mayden et al. 2009). Three datasets were constructed for analyses: (i) protein-coding gene (PCG) sequences of the complete mitogenome (dataset 1); (ii) PCG sequences of the complete mitogenome combined with nuclear DNA (ncDNA) (*Rag1*) sequences (dataset 2); and (iii) ncDNA (*Rag1*) sequences (dataset 3). All sequences were first aligned using MAFFT v. 7.475 (Katoh and Standley 2013), then trimmed using trimAl (Salvador et al. 2009). Maximum-likelihood (ML) and Bayesian-inference (BI) approaches were used to construct phylogenetic trees based on the three datasets. The ML analysis was performed using IQ-TREE v. 2.1.4 (Minh et al. 2020) based on the best-substitution model selected by ModelFinder in the IQ-TREE package (Kalyaanamoorthy et al. 2017). Nodal support was assessed based on 1,000 bootstrap replicates (Felsenstein 1985). The BI analysis was performed using MrBayes v. 3.2.7 (Ronquist et al. 2012), with the best-fit nucleotide substitution model also determined using ModelFinder. Four chains (three hot, one cold) were run for 5 million generations, with tree sampling every 1,000 generations and the first 25% of samples discarded as burn-in. Convergence was confirmed by ascertaining that the average standard deviation of split frequencies was below 0.01. The phylogenetic trees were viewed and edited using FigTree v. 1.4.4 (Rambaut 2014).

## ﻿Results

### ﻿Mitogenome composition and organization

**Figure 1.**
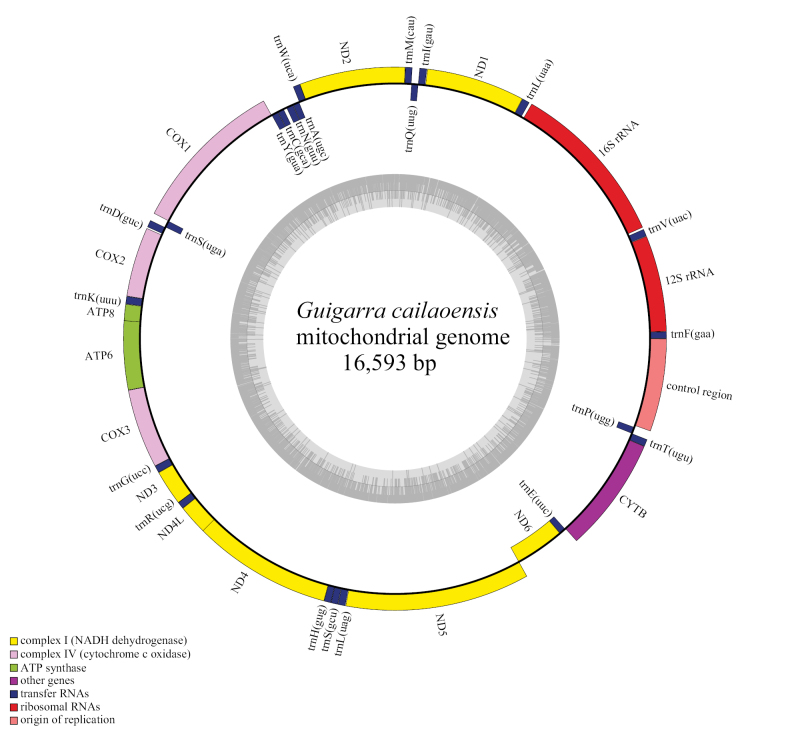
Circular map of complete mitogenome of *Guigarracailaoensis*.

The mitogenome of *Guigarracailaoensis* was identified as a circular double-stranded DNA sequence of 16,593 base pairs (bp) in length and included 13 protein-coding genes, 22 tRNA genes, two ribosomal RNA (rRNA) genes, and a putative control region (Table 1, Fig. 1). The base composition of *G.cailaoensis* was A = 32.2%, G = 15.5%, T = 26.4%, and C = 26.0%, with higher AT content (58.6%) than GC content (41.4%) (Table 2).

**Table 1.** Summary of genetic components of *Guigarracailaoensis* mitogenome.

Gene	Type	Initial bp	Final bp	Length	Direction	Strand	Start codon	Stop codon	Anticodon
*trnF*	tRNA	1	69	69	forward	H			GAA
*12S rRNA*	rRNA	70	1021	952	forward	H			
*trnV*	tRNA	1024	1095	72	forward	H			TAC
*16S rRNA*	rRNA	1115	2755	1641	forward	H			
*trnL1*	tRNA	2781	2856	76	forward	H			TAA
*ND1*	CDS	2858	3832	975	forward	H	ATG	TAA	
*trnI*	tRNA	3837	3908	72	forward	H			GAT
*trnQ*	tRNA	3907	3977	71	reverse	L			
*trnM*	tRNA	3979	4047	69	forward	H			CAT
*ND2*	CDS	4048	5094	1047	forward	H	ATG	TAG	
*trnW*	tRNA	5093	5163	71	forward	H			TCA
*trnA*	tRNA	5166	5234	69	reverse	L			TGC
*trnN*	tRNA	5236	5308	73	reverse	L			GTT
OL	rep_origin	5311	5342						
*trnC*	tRNA	5342	5407	66	reverse	L			GCA
*trnY*	tRNA	5409	5479	71	reverse	L			GTA
*COX1*	CDS	5481	7031	1551	forward	H	GTG	TAA	
*trnS1*	tRNA	7032	7102	71	reverse	L			GCT
*trnD*	tRNA	7106	7177	72	forward	H			GTC
*COX2*	CDS	7191	7881	691	forward	H	ATG	T--	
*trnK*	tRNA	7882	7957	76	forward	H			TTT
*ATP8*	CDS	7959	8123	165	forward	H	ATG	TAG	
*ATP6*	CDS	8117	8800	684	forward	H	ATG	TAA	
*COX3*	CDS	8800	9585	786	forward	H	ATG	TAA	
*trnG*	tRNA	9585	9656	72	forward	H			TCC
*ND3*	CDS	9657	10007	351	forward	H	ATG	TAG	
*trnR*	tRNA	10006	10075	70	forward	H			TCG
*ND4L*	CDS	10076	10372	297	forward	H	ATG	TAA	
*ND4*	CDS	10366	11746	1381	forward	H	ATG	T--	
*trnH*	tRNA	11747	11815	69	forward	H			GTG
*trnS2*	tRNA	11816	11884	69	forward	H			TGA
*trnL2*	tRNA	11886	11958	73	forward	H			TAA
*ND5*	CDS	11962	13785	1824	forward	H	ATG	TAA	
*ND6*	CDS	13782	14303	522	reverse	L	ATG	TAA	
*trnE*	tRNA	14304	14372	69	reverse	L			TTC
*CYTB*	CDS	14377	15517	1141	forward	H	ATG	T--	
*trnT*	tRNA	15518	15589	72	forward	H			TGT
*trnP*	tRNA	15589	15658	70	reverse	L			TGG
D-loop	D-loop	15676	16593	918	forward	H			

### ﻿PCGs and codon usage

The PCGs had a total length of 11,412 bp, accounting for 68.78% of the total length of the complete mitogenome. The *ND5* coding DNA sequence (CDS) had the highest number of base pairs (1 824 bp), while ATPase8 had the lowest (165 bp). The base percentage composition revealed a lower G + C content (41.1%) compared to the A + T content (58.9%). All PCGs were encoded on the heavy (H) strand, except for the *ND6* gene, which was encoded on the light (L) strand. All PCGs were initiated with the methionine codon ATG, except for *COX1*, which was initiated with GTG, consistent with previous labeonine mitochondrial DNAs (Wang et al. 2019). Two types of stop codon were identified: TAA (*ATP6*, *COX1*, *COX3*, *ND1*, *ND4L*, *ND5*, and *ND6*) and TAG (*ATP8*, *ND2*, and *ND3*). Incomplete stop codons were detected for *COX2*, *CYTB*, and *ND4* (Table 1).

**Figure 2.**
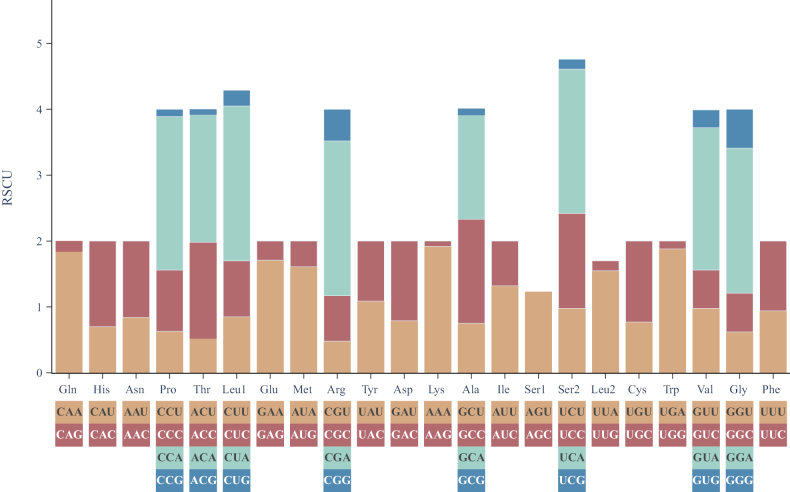
Relative synonymous codon usage (RSCU) in mitogenomes of *Guigarracailaoensis*.

The RSCU results indicated that six codons, CUA (2.35%), CGA (2.35%), CCA (2.33%), GGA (2.20%), UCA (2.19%), and GUA (2.16%), were the most frequently used. Additionally, the amino acids Pro, Thr, Leu1, Arg, Ala, Ser2, Val, and Gly were encoded by four codons, while all the other amino acids were encoded by two codons (Fig. 2).

### ﻿Ribosomal and transfer RNA genes

The *12S rRNA* and *16S rRNA* were 952 and 1,641 bp in length, respectively. They were located between *trnF* and *trnL1*, separated by *trnV*. The nucleotide composition of the rRNAs was A = 35.4%, C = 23.8%, G = 20.6%, and T = 20.2%. Thus, *G.cailaoensis* displayed a higher percentage of AT (55.6%) than GC (44.4%) (Table 2).

**Table 2.** Base composition and skewness of the mitogenome of *Guigarracailaoensis*.

Regions	Size (bp)	T(U)	C	A	G	AT (%)	GC (%)	AT skewness	GC skewness
*ATP6*	684	30.7	25.3	31.3	12.7	62.0	38.0	0.009	−0.331
*ATP8*	165	27.3	26.1	35.2	11.5	62.5	37.6	0.126	−0.387
*COX1*	1551	30.0	25.4	27.6	17.0	57.6	42.4	−0.043	−0.199
*COX2*	691	26.9	25.9	30.7	16.5	57.6	42.4	0.065	−0.222
*COX3*	786	27.2	28.0	29.0	15.8	56.2	43.8	0.032	−0.279
*CYTB*	1141	28.9	26.7	30.9	13.5	59.8	40.2	0.032	−0.329
*ND1*	975	27.9	26.9	31.4	13.8	59.3	40.7	0.059	−0.320
*ND2*	1047	24.4	30.2	33.2	12.2	57.6	42.4	0.154	−0.423
*ND3*	351	30.2	27.9	27.6	14.2	57.8	42.1	−0.044	−0.324
*ND4*	1381	27.9	26.4	32.4	13.3	60.3	39.7	0.075	−0.330
*ND4L*	297	29.3	27.9	27.3	15.5	56.6	43.4	−0.036	−0.287
*ND5*	1824	27.0	27.7	33.1	12.1	60.1	39.8	0.101	−0.392
*ND6*	522	42.7	11.7	15.3	30.3	58.0	42.0	−0.472	0.443
PCGs	11412	28.6	26.3	30.3	14.7	58.9	41.1	0.028	−0.282
rRNAs	2593	20.2	23.8	35.4	20.6	55.6	44.4	0.274	−0.073
tRNAs	1562	27.2	20.7	29.0	23.1	56.2	43.8	0.032	0.056
CR	918	34.0	18.7	33.9	13.4	67.9	32.1	−0.002	−0.166
Full	16593	26.4	26.0	32.2	15.5	58.6	41.5	0.100	−0.254

**Figure 3.**
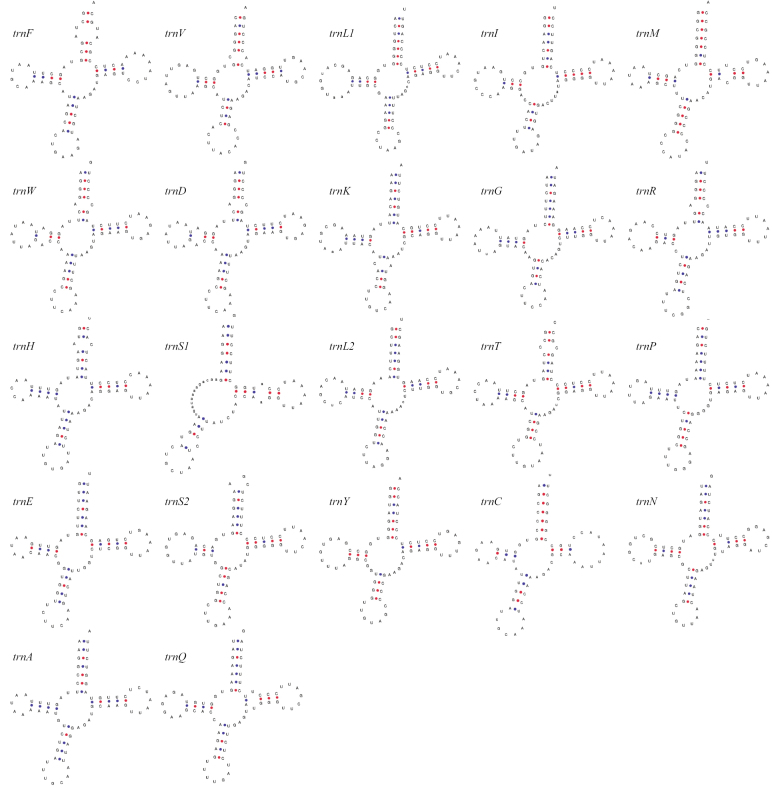
Secondary structures of 22 tRNA genes in *Guigarracailaoensis*.

Twenty-two tRNA genes were identified in *G.cailaoensis* mitogenome, including two for *trnL* and *trnS*, and one for each of the other amino acids (Table 1). Of these, 21 tRNA genes exhibited the typical cloverleaf secondary structure with four domains, while the *trnS1* gene lacked the D domain (D-stem and D-loop) (Fig. 3).

Fourteen tRNAs were encoded on the H-strand, while the remaining tRNAs were encoded on the L-strand (*trnQ*, *trnA*, *trnN*, *trnC*, *trnY*, *trnS1*, *trnE*, and *trnP*; Table 1). The length of these tRNAs varied, ranging from 66 bp (*trnC*) to 76 bp (*trnL1* and *trnK*), with a total length of 1,562 bp and accounting for 9.41% of the total mitogenome. Nucleotide composition of the tRNAs was A = 29.0%, C = 20.7%, G = 23.1%, and T = 27.2%, showing a higher AT content (56.2%) than GC content (43.8%) (Table 2).

### ﻿Non-coding region

The non-coding control region in the mitogenome, identified as the D-loop, was located between the *trnP* and *trnF* genes (Fig. 1). Spanning 918 bp in length, the region accounted for 5.53% of the whole mitogenome. The region exhibited a higher AT content (67.9%) than GC content (32.1%), with a nucleotide composition of A = 33.9%, T = 34.0%, C = 18.7%, and G = 13.4% (Table 2).

### ﻿Phylogenetic analysis

**Figure 4.**
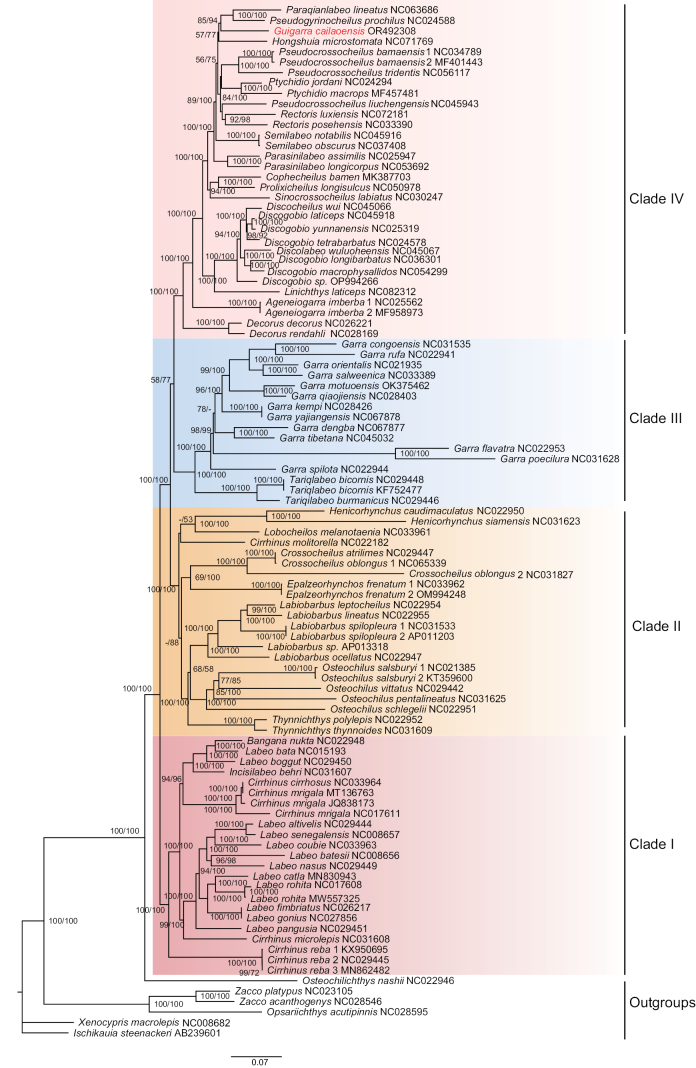
Phylogenetic tree of *Guigarracailaoensis* and 98 species downloaded from GenBank based on PCG sequences of complete mitogenomes (dataset 1). Nodal numbers are ML bootstrap values and BI posterior probability values, respectively. Only values above 50% are given.

**Figure 5.**
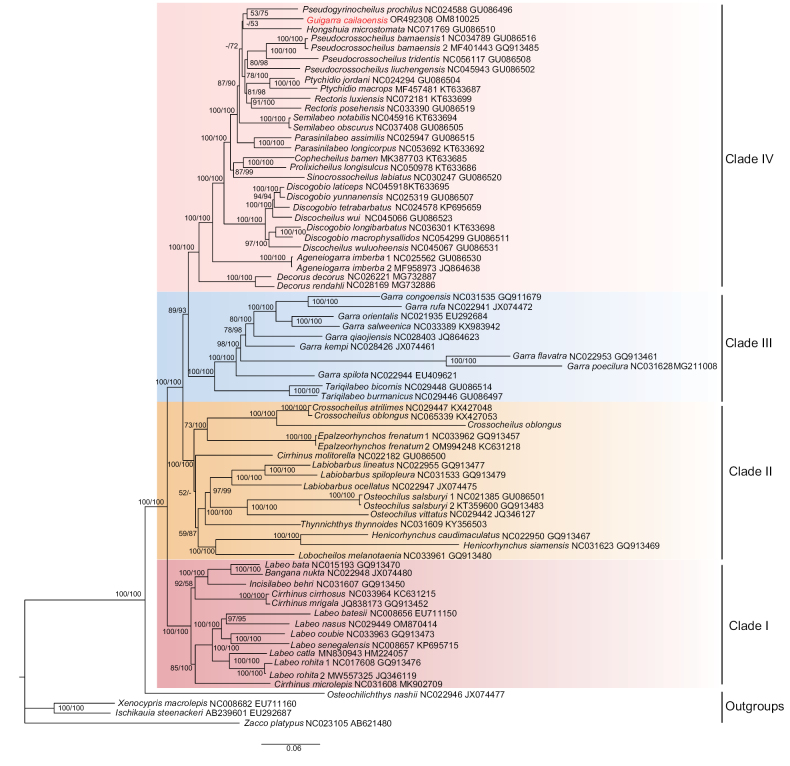
Phylogenetic tree of *Guigarracailaoensis* and 72 species downloaded from GenBank based on PCG sequences of complete mitogenome combined with ncDNA (*Rag1*) sequences (dataset 2). Nodal numbers are ML bootstrap values and BI posterior probability values, respectively. Only values above 50% are given.

**Figure 6.**
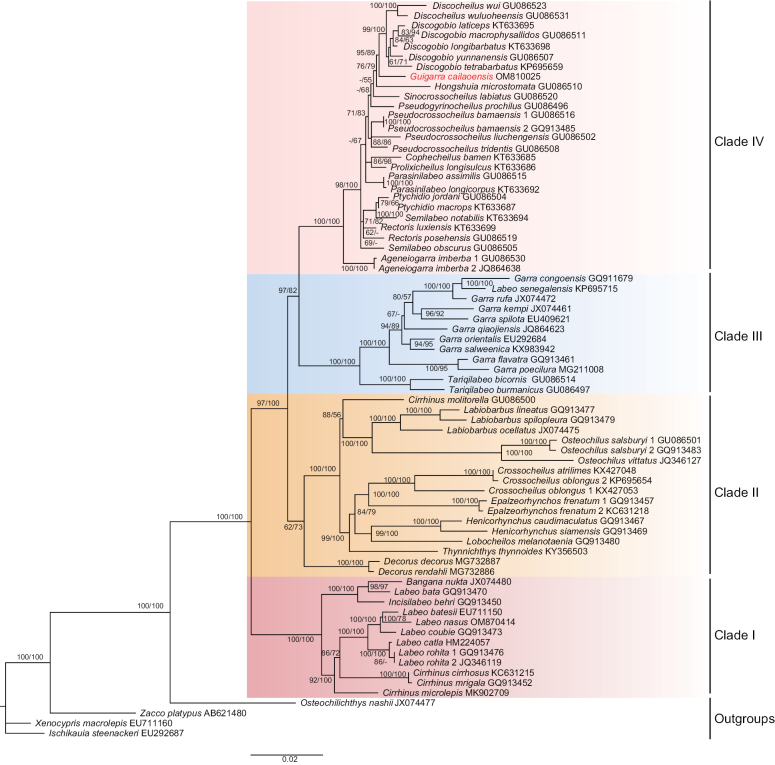
Phylogenetic tree of *Guigarracailaoensis* and 72 species downloaded from GenBank based on ncDNA (*Rag1*) sequences (dataset 3). Nodal numbers are ML bootstrap values and BI posterior probability values, respectively. Only values above 50% are given.

The best-fit models for ML and BI analyses were identified, as shown in Table 3. Phylogenetic trees derived from dataset 1 and 2 were remarkably similar, while those from dataset 3 showed slight variations. Within each dataset, the trees generated from ML and BI analyses were consistent across all taxa, differing only slightly in their support values. Consequently, the ML trees were presented here together with the nodal support values generated by ML and BI analyses, respectively. Notably, all taxa within Labeoninae were recovered as a monophyletic clade, further subdivided into four major lineages. In dataset 1, *G.cailaoensis* formed a sister taxon to the lineage consisting of *Paraqianlabeolineatus* and *Pseudogyrinocheilusprochilus*, and in dataset 2 formed a sister taxon to *Pseudogyrinocheilusprochilus*. However, in dataset 3, *G.cailaoensis* formed a sister taxon to the lineage consisting of *Discogobio* and *Discocheilus* (Figs 4–6).

**Table 3.** The best-fit models selected by ModelFinder for three datasets.

	ML	BI
Dataset 1 (Mitogenome)	TIM2 + F + R6	GTR + F + I + G4
Dataset 2 (Mitogenome+*Rag1*)	GTR + F + R6	GTR + F + I + G4
Dataset 3 (*Rag1*)	TIM2e + I + G4	SYM + I + G4

## ﻿Discussion

The mitochondrial gene structure in *Guigarracailaoensis* is congruent with that of other vertebrate animals, consisting of double-stranded circular DNA spanning approximately 15–20 kb (Miya et al. 2001). Furthermore, its base composition is also consistent with results observed in other Labeoninae fish species (Zheng and Yang 2017; Zhang et al. 2023), and the features of its tRNA genes are consistent with those observed in metazoan mitochondrial DNA (Watanabe et al. 2014).

Our phylogenetic analyses of the subfamily Labeoninae across three datasets identified four lineages, which is consistent with the results of Yang et al. (2012). When considering the species common to all three datasets, the phylogenetic trees derived from datasets 1 and 2 were nearly identical, while those derived from dataset 3 differed slightly. The main divergence was observed in the placement of the *Decorusdecorus* and *Decorusrendahli* lineage, located in Clade IV in datasets 1 and 2 but in Clade II in dataset 3. *Guigarracailaoensis* was positioned in Clade IV, corresponding to the fourth clade described by Yang et al. (2012) and within the karst group defined by Zheng et al. (2016). Currently, approximately eight genera within Labeoninae are characterized by the presence of an oral disc on the lower lip, including *Ageneiogarra*, *Ceratogarra*, *Discocheilus*, *Discogobio*, *Garra*, *Guigarra*, *Sinigarra*, and *Placocheilus* (Wang et al. 2022). Our results revealed that the genera with oral discs are distributed across different lineages in the three datasets. Although *G.cailaoensis* possesses an oral disc on its lower lip, it was not closely related to other oral disc-bearing species based on datasets 1 and 2. In dataset 1, it is closely related to *Paraqianlabeolineatus* and *Pseudogyrinocheilusprochilus*, and in dataset 2, it is closely related to *Pseudogyrinocheilusprochilus*, neither of which possess an oral disc on the lower lip. The results derived from dataset 1 and 2 are essentially consistent, because only the complete mitogenome sequences of *Pseudogyrinocheilusprochilus* are available in dataset 1 and 2, and *Paraqianlabeolineatus* was not included in dataset 2. However, in dataset 3, *G.cailaoensis* is closely related to the lineage consisting of *Discogobio* and *Discocheilus*, both of which possess an oral disc on the lower lip. The phylogenetic position of *G.cailaoensis* and its closely related taxa derived from dataset 3 are consistent with the results of Wang et al. (2022), who reported a close affinity between *G.cailaoensis*, *Discogobio*, and *Discocheilus* based on one nuclear and two mitochondrial genes. We hypothesize that the observed inconsistencies among the results of different datasets likely stem from differences in the phylogenetic signal contribution between mitogenome and nuclear gene loci, as discussed by Zheng et al. (2010). Nonetheless, a variety of species with an oral disc on the lower lip were clustered into different lineages in the results from three datasets that may indicate that the development of the oral disc is homoplastic within the subfamily Labeoninae. In conclusion, the successful assembly of the complete mitogenome of *G.cailaoensis* not only enhances our understanding of its genetic background but may also prove valuable for conservation and resource restoration strategies in the area.

## ﻿Additional information

### Author ORCID

Lan-Ping Zheng ￼ https://orcid.org/0000-0002-9855-6503

### Data availability

All of the data that support the findings of this study are available in the main text. The genome sequence data are openly available in GenBank of NCBI at (https://www.ncbi.nlm.nih.gov/) under the accession no. OR492308.
